# A clinical–radiomics model based on noncontrast computed tomography to predict hemorrhagic transformation after stroke by machine learning: a multicenter study

**DOI:** 10.1186/s13244-023-01399-5

**Published:** 2023-03-29

**Authors:** Huanhuan Ren, Haojie Song, Jingjie Wang, Hua Xiong, Bangyuan Long, Meilin Gong, Jiayang Liu, Zhanping He, Li Liu, Xili Jiang, Lifeng Li, Hanjian Li, Shaoguo Cui, Yongmei Li

**Affiliations:** 1 Department of Radiology, The First Affiliated Hospital of Chongqing Medical University, No. 1 Youyi Road, Yuzhong District, Chongqing, 400016 China; 2 Department of Radiology, Chongqing General Hospital, Chongqing, China; 3 College of Computer and Information Science, Chongqing Normal University, No. 37, Middle University Town Road, Shapingba District, Chongqing, 400016 China; 4 Department of Radiology, Haikou Affiliated Hospital of Central South University Xiangya School of Medicine, Haikou, China; 5 Department of Radiology, People’s Hospital of Yubei District of Chongqing City, Chongqing, China; 6 Department of Radiology, The Second People’s Hospital of Hunan Province/Brain Hospital of Hunan Province, Changsha, China; 7 Department of Radiology, Changsha Central Hospital (The Affiliated Changsha Central Hospital, Hengyang Medical School, University of South China), Changsha, China; 8 Department of Radiology, The First Affiliated Hospital of Hainan Medical University, Haikou, China

**Keywords:** Acute ischemic stroke, Hemorrhagic transformation, Noncontrast computed tomography, Radiomics, Machine learning

## Abstract

**Objective:**

To build a clinical–radiomics model based on noncontrast computed tomography images to identify the risk of hemorrhagic transformation (HT) in patients with acute ischemic stroke (AIS) following intravenous thrombolysis (IVT).

**Materials and methods:**

A total of 517 consecutive patients with AIS were screened for inclusion. Datasets from six hospitals were randomly divided into a training cohort and an internal cohort with an 8:2 ratio. The dataset of the seventh hospital was used for an independent external verification. The best dimensionality reduction method to choose features and the best machine learning (ML) algorithm to develop a model were selected. Then, the clinical, radiomics and clinical–radiomics models were developed. Finally, the performance of the models was measured using the area under the receiver operating characteristic curve (AUC).

**Results:**

Of 517 from seven hospitals, 249 (48%) had HT. The best method for choosing features was recursive feature elimination, and the best ML algorithm to build models was extreme gradient boosting. In distinguishing patients with HT, the AUC of the clinical model was 0.898 (95% CI 0.873–0.921) in the internal validation cohort, and 0.911 (95% CI 0.891–0.928) in the external validation cohort; the AUC of radiomics model was 0.922 (95% CI 0.896–0.941) and 0.883 (95% CI 0.851–0.902), while the AUC of clinical–radiomics model was 0.950 (95% CI 0.925–0.967) and 0.942 (95% CI 0.927–0.958) respectively.

**Conclusion:**

The proposed clinical–radiomics model is a dependable approach that could provide risk assessment of HT for patients who receive IVT after stroke.

**Supplementary Information:**

The online version contains supplementary material available at 10.1186/s13244-023-01399-5.

## Key points

- Noncontrast computed tomography (NCCT) is valuable for predicting hemorrhagic transformation (HT) after intravenous thrombolysis (IVT) treatment.
- Machine learning is vital for predicting HT.
- NCCT radiomics integrated with clinical factors could facilitate predicting HT.

**Supplementary Information:**

The online version contains supplementary material available at 10.1186/s13244-023-01399-5.

## Introduction

Acute ischemic stroke (AIS), which has significant death and disability rates, poses a serious threat to human life [1]. The rapid intravenous delivery of recombinant tissue type plasminogen activator remains the main method for the early management of AIS in selected individuals [2].

Patients with stroke undergoing intravenous thrombolysis (IVT) are at risk of developing hemorrhagic transformation (HT) [3]. Between 10 and 48% of patients who receive thrombolytic therapy develop HT [4]. In cases of HT following thrombolysis, the prognosis is worse, regardless of whether the condition is symptomatic hemorrhagic transformation or not. This could also affect subsequent treatment. Moreover, with the trend of extending the time window further, it is important to detect HT development earlier and more precisely. Therefore, stroke neurologists can take proactive measures to prevent clinical deterioration and make optimal treatment decisions if HT is predicted early.

At present, various methods can be used to predict HT, for example, clinical indicators [5–7], imaging data [8–14] or combination of clinical features and imaging markers [15]. However, some previous studies involved fewer predictors, and the effectiveness of the prediction model was relatively limited (AUC < 0.75). Moreover, some studies have applied magnetic resonance imaging (MRI), which is time-consuming. In addition, because computed tomography perfusion examinations generated much more radiation and were expensive, they cannot be performed in smaller medical centers. Nevertheless, noncontrast computed tomography (NCCT) images were chosen as the study’s source images because they are more frequently performed, take less time, and, most importantly, are recommended by the guidelines for AIS [2]. NCCT images, particularly for thick images with slice thickness of 5 mm and slice spacing of 5 mm, are also much more practical for reexamination.

Radiomics analysis, a new method for assisting precision medicine, can automatically extract radiomics features from medical images, which is anticipated to overcome the shortcomings of visual image evaluation [16, 17]. According to previous studies, NCCT-based radiomics features have superior abilities in various disciplines. Regarding stroke, several problems have been reported, such as prediction of hemorrhage expansion after spontaneous intracerebral hemorrhage [18, 19], early identification of ischemic stroke [20] and estimating infarction onset time [21]. However, the method of obtaining radiomics features based on NCCT has not been utilized for the prediction of HT risk following thrombolysis, which is the primary focus of the present study.

Numerous machine learning (ML)-related studies have emerged in recent years, and ML undeniably performs quite well in classification and prediction [22]. At present, stroke-related ML research has been reported, and its performance is very outstanding [23]. Some studies have found that the method of predicting HT by ML is feasible [24, 25]. In this study, we developed and validated ML algorithms to automatically predict HT in patients with AIS receiving IVT combining clinical and radiomics-based features.

## Materials and methods

### Patient selection

This multicenter retrospective analysis was approved by the institutional review boards of our hospital, and the necessity for patient informed consent was waived.

Clinical data and NCCT images were collected from seven hospitals from June 2012 to December 2021. A total of 822 consecutive patients with AIS were chosen for inclusion. This study included patients with AIS who met the following criteria: (1) were undergoing IVT in accordance with the management guidelines for AIS, (2) had completed NCCT examination before IVT therapy, and (3) underwent a follow-up MRI or NCCT within 36 h after receiving IVT. Patients with head trauma injuries, primary cerebral hemorrhage or brain tumors, hemorrhagic infarction upon admission, insufficient data, and severe artifacts on NCCT images were excluded.

**Fig. 1:**
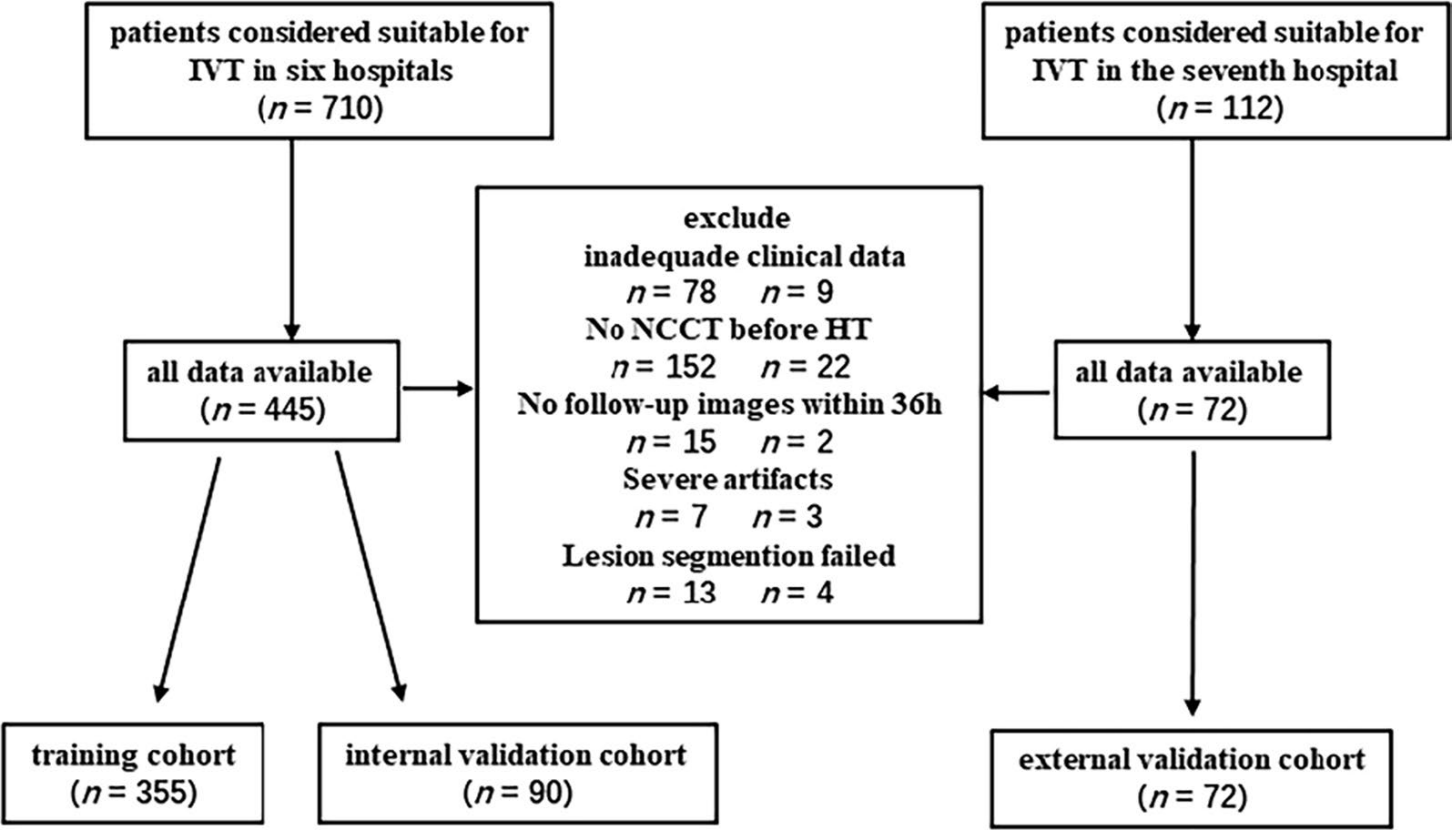
Flowchart of patients’ selection (IVT, intravenous thrombolysis; HT, hemorrhagic transformation)

Finally, a total of 517 patients (282 patients without HT and 235 patients with HT) were enrolled. The dataset from six hospitals (the First Affiliated Hospital of Chongqing Medical University, Chongqing General Hospital, Haikou Affiliated Hospital of Central South University Xiangya School of Medicine, the Second People’s Hospital of Hunan Province/Brain Hospital of Hunan Province, the First Affiliated Hospital of Hainan Medical University, Changsha Central Hospital (the Affiliated Changsha Central Hospital, Hengyang Medical School, University of South China)) was randomly divided into training cohort (n = 355) and internal validation cohort (n = 90). Data from the seventh hospital (People's Hospital of Yubei District of Chongqing City), which included 33 patients with HT and 39 patients without HT, were kept as an independent external validation cohort. The flowchart of patients’ preparation is depicted in Fig. 1.

### Obtaining clinical data

Clinical data (demographic data and laboratory tests) were obtained. Laboratory tests on admission (including blood pressure, blood glucose levels, and blood lipid levels), initial National Institute of Health Stroke Scale (NIHSS) score, onset-to-CT time, medical history (including smoking (smoking index), drinking (drinking index), previous stroke, diabetes mellitus, and atrial fibrillation) and Trial of ORG 10172 in acute stroke treatment (TOAST) typing of acute stroke etiology were examined separately from the electronic medical record system.

### Imaging acquisition

Additional file 1: Table S1 presents the models of CT scanners and scanning parameters used in seven institutions (Additional file).

### Reference standard

HT was determined based on the European Co-operative Acute Stroke Study-II trial [26]. CT images show high-density lesions, including hemorrhagic infarction (HI) and parenchymal hemorrhage (PH). In this study, two neuroradiology staff members independently evaluated HT on follow-up NCCT or MRI within 36 h following IVT therapy for all the training and testing datasets without knowledge of the patient outcome (X.H. and L.B.Y., directors with 10 years of experience in neuroradiology). Any discrepancy was resolved by consensus. By comparing prior CT or MRI images, HT and contrast agent extravasation could be differentiated, and the conclusion was supported by examination performed 2–7 days following treatment.

### Data preprocessing

Clinical data were processed using Z-score normalization after missing values were filled in using K-nearest neighbor (KNN). Furthermore, the steps of NCCT image normalization were as follows: (a) every NCCT image slice was resampled to a unified pixel dimension size of 1.0 × 1.0 × 1.0 mm^3^; (b) image intensity of every NCCT image was normalized by the gray-level discretization method with a fixed number of bins (256 bins); The purpose of the two steps was to minimize any potential effects brought on by scanners, scanning parameters. In addition, NCCT images were set in a fixed head window (window level = 50 Hounsfield unit (Hu); window width = 110 Hu). The purpose was to ensure there was less difference while manually drawing lesions.

### Radiomics analysis

The region of interest (ROI) of cerebral infarction was manually defined on the axial slices of NCCT images using the 3D-Slicer software, slice by slice, around its perimeter. If the lesion’s border was not clearly visible on the NCCT image, diffusion-weighted images taken within 6 h were used to draw the border.

To ensure the reproducibility of radiomics features, the intra- and interobserver correlation coefficients were computed using the ROIs randomly selected from 20 patients. By comparing the ROIs’ features of radiologists 1, the intraclass correlation coefficient (ICC) was determined (twice, one month apart). Comparing the ROIs’ features of radiologists 1 and 2 allowed for the calculation of the inter-ICC. The features (with both ICCs threshold ≥ 0.95) having good reliability were added to the subsequent analysis (Additional file 1: Figure S1).

By applying the mask of ROIs, radiomics features were extracted based on the 3D-Slicer package (Version no. 4.13.0) (https://www.slicer.org/). Eight categories of radiomics features were obtained as follows: first order; shape; shape 2D; gray-level co-occurrence matrix (GLCM); gray-level run length matrix (GLRLM); gray-level size zone matrix (GLSZM); neighboring gray-tone difference matrix and gray-level dependence matrix.

### Finding the best method to select features

**Fig. 2:**
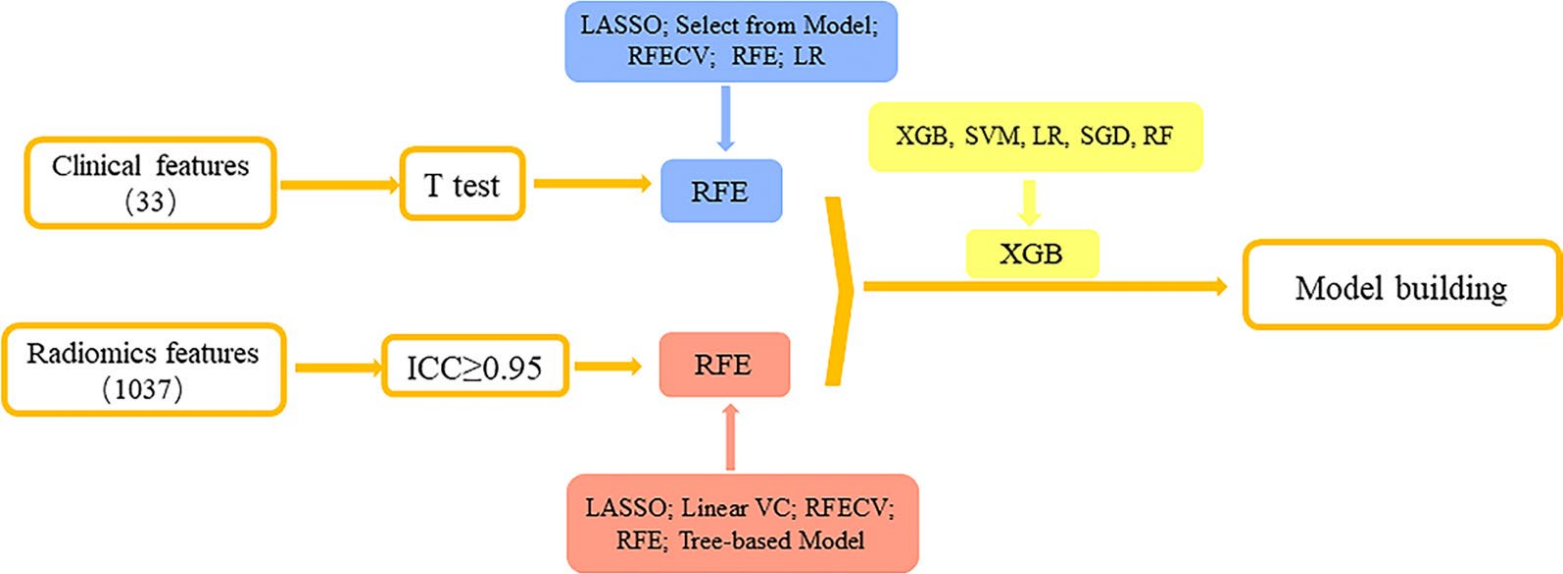
Flowchart of the most important features’ selection (The numbers in parentheses are characteristic numbers; ICC, Intercorrelation Coefficient; LASSO, Least Absolute Shrinkage and Selection Operator; RFECV, Recursive Feature Elimination Cross Validation; RFE, Recursive Feature Elimination; LR, Logistic Regression; Linear SVC, Linear Support Vector Classification; SGD, Stochastic Gradient Descent; SVM, Support Vector Machine; RF, Random Forest; XGB, eXtreme Gradient Boosting)

Firstly, for clinical data, T-test was used to test the characteristics of significant difference between HT group and non-HT group in the training cohort. Then we compared the five common dimensionality reduction methods (including Least Absolute Shrinkage and Selection Operator (LASSO), Select from Model, Recursive Feature Elimination Cross Validation (RFECV), Recursive Feature Elimination (RFE), and Logistic Regression (LR)) by ten-fold cross validation in the training cohort to choose the best one. And the best method was used to select the most important clinical features (the blue part of Fig. 2).

Secondly, for radiomics features, the unrepeatable features below the ICC threshold were eliminated from the 1037 radiomics features obtained after sketching, leaving 778 features. The best of the five popular methods (LASSO, Linear Support Vector Classification, RFECV, RFE, Tree-based Model) was chosen by ten-fold cross validation in the training cohort to select the most significant radiomics features (the red part of Fig. 2).

### Finding the best ML algorithm to build models

Before modeling, the effects of five ML algorithms (eXtreme Gradient Boosting (XGB), Support Vector Machine, LR, Stochastic Gradient Descent, and random forest) were compared to identify the best algorithm by ten-fold cross validation in the training cohort (the yellow part of Table 4) to develop the prediction model of HT.

### Building models

Independent clinical prediction factors (*p* < 0.05) for HT were obtained by the best dimensionality reduction method mentioned above (Fig. 2). Then, they were used to develop a clinical model by the best ML algorithm. And the model was used in the internal and external validation cohorts to test the efficiency.

Using the same process as developing the clinical model, a radiomics model was constructed in the training cohort utilizing the most significant radiomics features which were ultimately selected. In addition, the internal and external validation cohorts also need to verify the radiomics model.

Finally, the clinical–radiomics model, which combined distinct clinical risk variables and important radiomics features, was developed in the training cohort and then validated independently in the internal and external validation cohorts.

### Model evaluation

To assess each model’s performance, the receiver operating characteristic curve was created, and the AUC was calculated.

**Fig. 3:**
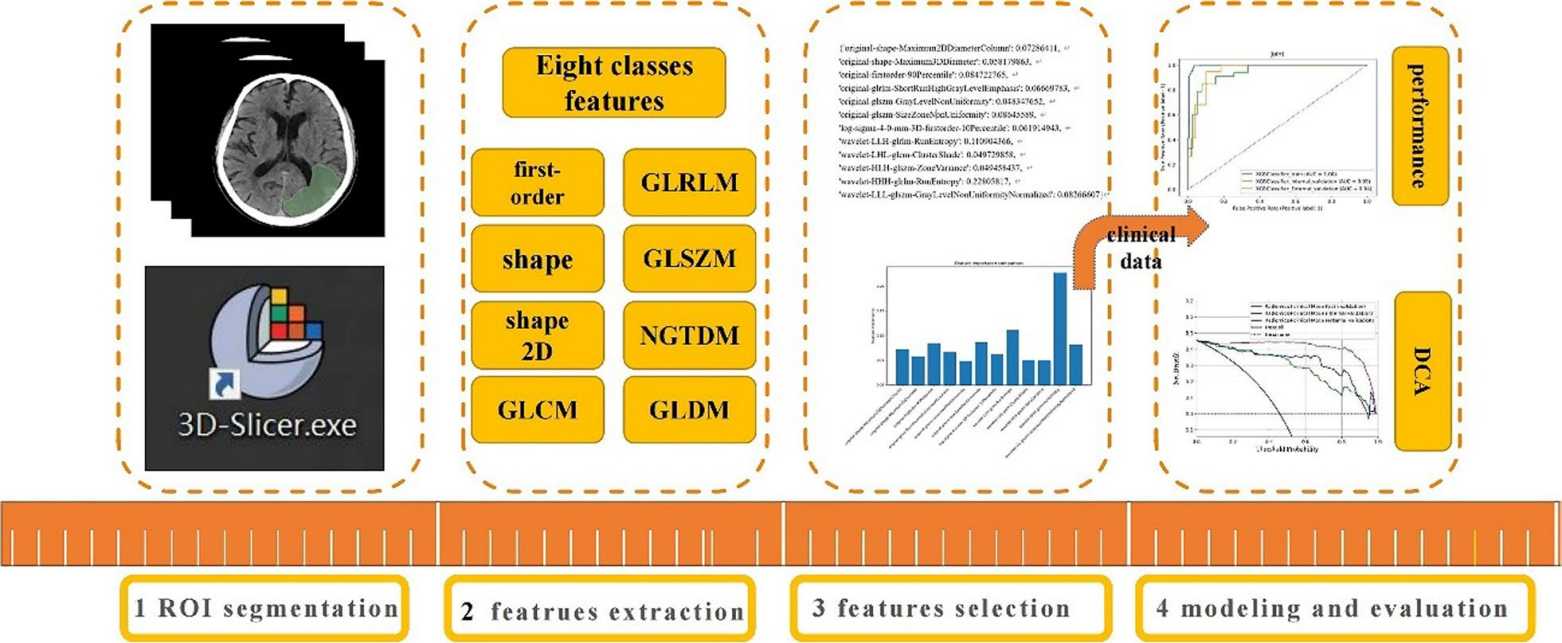
Workflow of the clinical–radiomics model of predicting HT after IVT (IVT, intravenous thrombolysis; HT, hemorrhagic transformation; DCA, decision curve analysis)

The calibration curve was presented to assess the model’s capacity for calibration, which compares the consistency between real results and the clinical–radiomics model. To assess the combined model’s clinical utility, a decision curve analysis (DCA) was used. The workflow of the radiomics analysis of the ROI and the building model is described in Fig. 3.

### Statistical analysis

All statistical data were analyzed using R software (version 4.1.3) (https://www.r-project.org/). Normal data were presented as means ± standard deviation, and qualitative data were shown as numbers and percentages. A chi-squared test, a two-sample t-test, or the Mann–Whitney U test was used to evaluate the clinical characteristics. To compare the AUCs of various models, the DeLong test was utilized. A two-sided *p* value < 0.05 was deemed significant for all statistical analyses.

## Results

### Patients’ clinical characteristics

This study comprised 517 individuals (333 male [64.4%], mean age ± standard deviation 67.02 ± 12.67) from seven hospitals. The clinical data of all patients are shown in Table 1.

**Table 1:** Characteristics of all the patients

Variables	Overall (n = 517)	Training cohort (n = 355)	Internal validation cohort (n = 90)	External validation cohort (n = 72)	*p*
*Gender, n (%)*					0.039
Male	333 (64.4)	234 (65.9)	62 (68.9)	37 (51.4)	
Female	184 (35.6)	121 (34.1)	28 (31.1)	35 (48.6)	
age, years, mean ± SD	67.02 ± 12.67	67.19 ± 12.39	66.83 ± 12.15	66.44 ± 14.70	0.891
Smoking index, mean ± SD	307.23 ± 633.55	325.51 ± 662.15	226.72 ± 428.54	317.78 ± 700.63	0.414
drinking index, mean ± SD	1270.36 ± 3193.83	1268.38 ± 3107.17	1443.89 ± 3768.48	1063.19 ± 2848.41	0.753
*Previous stroke, n (%)*					0.144
No	417 (80.7)	292 (82.3)	73 (81.1)	52 (72.2)	
Yes	100 (19.3)	63 (17.7)	17 (18.9)	20 (27.8)	
*Diabetes mellitus, n (%)*					0.192
No	368 (71.2)	258 (72.7)	57 (63.3)	53 (73.6)	
Yes	149 (28.8)	97 (27.3)	33 (36.7)	19 (26.4)	
*Atrial-fibrillation, n (%)*					0.193
No	410 (79.3)	289 (81.4)	66 (73.3)	55 (76.4)	
Yes	107 (20.7)	66 (18.6)	24 (26.7)	17 (23.6)	
OTC, hours, mean ± SD	5.43 ± 2.77	5.20 ± 2.49	5.68 ± 3.01	6.25 ± 3.51	0.008
SBP, mm Hg, mean ± SD	152.32 ± 24.41	153.45 ± 24.01	150.39 ± 27.32	149.18 ± 22.41	0.285
DBP, mm Hg, mean ± SD	86.97 ± 13.58	87.68 ± 13.03	85.48 ± 15.46	85.37 ± 13.67	0.217
Baseline NIHSS, mean ± SD	9.15 ± 7.17	9.18 ± 7.07	9.87 ± 7.91	8.09 ± 6.61	0.288
Hgb, g/L, mean ± SD	135.40 ± 53.00	136.38 ± 62.78	134.90 ± 17.77	131.21 ± 18.60	0.749
PLT, 10^9^/L, mean ± SD	209.67 ± 82.15	211.25 ± 84.67	210.68 ± 84.28	200.65 ± 65.56	0.604
WBC, 10^9^/L, mean ± SD	8.75 ± 3.77	8.65 ± 3.87	8.95 ± 3.52	8.99 ± 3.57	0.679
Neu, 10^9^/L, mean ± SD	6.47 ± 3.79	6.47 ± 4.08	6.80 ± 3.40	6.07 ± 2.60	0.483
Ly, 10^9^/L, mean ± SD	1.54 ± 1.15	1.49 ± 0.81	1.64 ± 2.01	1.66 ± 1.11	0.327
Mn, 10^9^/L, mean ± SD	0.59 ± 0.54	0.61 ± 0.63	0.58 ± 0.27	0.50 ± 0.16	0.27
N/M, mean ± SD	5.80 ± 6.19	5.81 ± 6.50	6.34 ± 6.30	5.08 ± 4.13	0.436
L/M, mean ± SD	3.28 ± 3.05	3.14 ± 2.65	3.42 ± 4.36	3.76 ± 2.86	0.266
Eon, 10^9^/L, mean ± SD	0.14 ± 0.25	0.13 ± 0.18	0.16 ± 0.27	0.19 ± 0.44	0.079
TC, mmol/L, mean ± SD	4.56 ± 1.20	4.62 ± 1.17	4.52 ± 1.36	4.32 ± 1.11	0.159
TG, mmol/L, mean ± SD	1.65 ± 1.29	1.63 ± 1.29	1.90 ± 1.61	1.49 ± 0.69	0.1
HDL, mmol/L, mean ± SD	1.23 ± 0.46	1.24 ± 0.49	1.21 ± 0.42	1.19 ± 0.38	0.606
LDL, mmol/L, mean ± SD	2.78 ± 0.97	2.83 ± 0.94	2.70 ± 1.10	2.62 ± 0.97	0.176
BUN, mmol/L, mean ± SD	6.35 ± 3.42	6.42 ± 3.62	6.34 ± 3.30	6.02 ± 2.46	0.666
Cr, μmol/L, mean ± SD	80.75 ± 49.30	83.96 ± 55.50	75.36 ± 33.78	71.66 ± 26.41	0.081
Glu, mmol/L, mean ± SD	7.88 ± 3.44	7.74 ± 3.30	8.55 ± 3.88	7.74 ± 3.47	0.13
HbA1c, %, mean ± SD	7.95 ± 6.31	8.19 ± 7.45	7.54 ± 2.09	7.33 ± 2.60	0.46
INR, mean ± SD	1.02 ± 0.15	1.01 ± 0.12	1.03 ± 0.18	1.05 ± 0.19	0.198
FDPs, μg/mL, mean ± SD	6.67 ± 12.96	7.00 ± 14.23	5.51 ± 8.57	6.53 ± 10.78	0.618
D-Dimer, mg/L, mean ± SD	2.53 ± 5.39	2.73 ± 6.03	2.29 ± 4.27	1.85 ± 2.52	0.403
NT-proBNP, mean ± SD	1062.22 ± 2025.72	1048.42 ± 2026.11	1192.05 ± 2145.51	967.99 ± 1885.10	0.763
*TOAST types, n (%)*					0.463
LAA	300 (58.0)	210 (59.2)	51 (56.7)	39 (54.2)	
CE	151 (29.2)	106 (29.9)	26 (28.9)	19 (26.4)	
SVO	48 (9.3)	29 (8.2)	8 (8.9)	11 (15.3)	
SOE	5 (1.0)	4 (1.1)	1 (1.1)	0 (0.0)	
SUE	13 (2.5)	6 (1.7)	4 (4.4)	3 (4.2)	

Categorical variables are represented by the number (percent), and continuous variables are represented by mean (± standard deviation)

OTC, onset-to-CT time; SBP, systolic blood pressure; DBP, diastolic blood pressure; NIHSS, National Institute of Health Stroke Scale; Hgb, Hemoglobin; PLT, Blood platelet; WBC, white blood cell; Neu, neutrophil; Ly, lymphocyte; Mn, monocyte; N/M, neutrophil-monocyte ratio; L/M, lymphocyte-monocyte ratio; Eon, eosinophils; TC, total cholesterol; TG, triglycerides; HDL, high-density lipoprotein cholesterol; LDL, low-density lipoprotein cholesterol; BUN, blood urea nitrogen; Cr, Creatinine; Glu, Glucose; HbA1c, glycated hemoglobin A1c; INR, international normalized ratio; FDPs, fibrin degradation products; NT-proBNP, *N*-terminal pro-brain natriuretic peptide, ng/L; LAA, large artery atherosclerosis; SVO, small vessel occlusion; CE, cardioembolism; SOE, stroke of other determined etiology; SUE, stroke of undetermined etiology

### Outcomes of screening features

According to Tables 2 and 3, the RFE is the best method to select features for clinical and radiomics data. This study found that XGB had the highest accuracy and AUC (Table 4).

**Table 2:** Performance of five methods to select clinical features in the training cohort

Selecting method	Dim	AUC	ACC	SEN	SPE	PPV	NPV
LASSO	15	0.900	0.822	0.859	0.792	0.776	0.870
SelectFromModel	10	0.892	0.829	0.834	0.825	0.800	0.856
RFECV	15	0.915	0.826	0.856	0.798	0.781	0.871
RFE	5	0.917	0.832	0.815	0.846	0.818	0.844
LR	13	0.903	0.827	0.842	0.814	0.793	0.860

LASSO, Least Absolute Shrinkage and Selection Operator; RFECV, Recursive Feature Elimination Cross Validation; RFE, Recursive Feature Elimination; LR, Logistic Regression; Dim, dimension; AUC, area under the receiver operator characteristic curve; ACC, accuracy; SEN, sensitivity; SPE, specificity; PPV, positive predictive value; NPV, negative predictive value

**Table 3:** Performance of five methods to select radiomics features in the training cohort

Selecting method	Dim	AUC	ACC	SEN	SPE	PPV	NPV
LASSO	41	0.931	0.823	0.788	0.853	0.819	0.829
Linear SVC	19	0.917	0.831	0.805	0.853	0.823	0.840
RFECV	116	0.936	0.840	0.810	0.865	0.836	0.845
RFE	12	0.911	0.832	0.802	0.857	0.825	0.839
Tree-based model	12	0.865	0.778	0.720	0.827	0.777	0.779

LASSO, Least Absolute Shrinkage and Selection Operator; Linear SVC, Linear Support Vector Classification; RFECV, Recursive Feature Elimination Cross Validation; RFE, Recursive Feature Elimination; Dim, dimension; AUC, area under the receiver operator characteristic curve; ACC, accuracy; SEN, sensitivity; SPE, specificity; PPV, positive predictive value; NPV, negative predictive value

**Table 4:** Performance of five machine learning algorithms to predict HT in the training cohort

	Training cohort					
	AUC	ACC	SEN	SPE	PPV	NPV
SGD	0.912	0.854	0.883	0.831	0.818	0.896
SVM	0.936	0.870	0.942	0.810	0.806	0.943
LR	0.874	0.813	0.832	0.798	0.775	0.850
RF	0.926	0.838	0.800	0.869	0.837	0.839
XGB	0.953	0.894	0.895	0.894	0.876	0.911

HT, hemorrhagic transformation; SGD, Stochastic Gradient Descent; SVM, Support Vector Machine; LR, Logistic Regression; RF, Random Forest; XGB, eXtreme Gradient Boosting; AUC, area under the receiver operator characteristic curve; ACC, accuracy; SEN, sensitivity; SPE, specificity; PPV, positive predictive value; NPV, negative predictive value

The result of the univariate analysis for clinical risk factors associated with HT in the training cohort is presented in Table 5. After RFE, five independent risk factors were selected, including baseline NIHSS (0.379), fibrin degradation products (FDPs) (0.183), monocyte (0.147), D-dimer (0.164), and N-terminal pro-brain natriuretic peptide (NT-proBNP) (0.128) (the number reflects the importance of each feature).

**Table 5:** Univariate analysis for HT in the training cohort

Variables	Overall (n = 355)	Non-HT (n = 194)	HT (n = 161)	*p*
*Gender, n (%)*				0.555
Male	234 (65.9)	131 (67.5)	103 (64.0)	
Female	121 (34.1)	63 (32.5)	58 (36.0)	
age, years, mean ± SD	67.19 ± 12.39	67.14 ± 12.31	67.24 ± 12.53	0.941
Smoking index, mean ± SD	325.51 ± 662.15	310.00 ± 485.00	344.19 ± 828.06	0.629
drinking index, mean ± SD	1268.38 ± 3107.17	1436.73 ± 3366.58	1065.53 ± 2759.25	0.263
*Previous stroke, n (%)*				0.001
No	292 (82.3)	172 (88.7)	120 (74.5)	
Yes	63 (17.7)	22 (11.3)	41 (25.5)	
*Diabetes mellitus, n (%)*				0.119
No	258 (72.7)	148 (76.3)	110 (68.3)	
Yes	97 (27.3)	46 (23.7)	51 (31.7)	
*Atrial-fibrillation, n (%)*				0.001
No	289 (81.4)	171 (88.1)	118 (73.3)	
Yes	66 (18.6)	23 (11.9)	43 (26.7)	
OTC, hours, mean ± SD	5.20 ± 2.49	5.70 ± 2.66	4.61 ± 2.12	< 0.001
SBP, mm Hg, mean ± SD	153.45 ± 24.01	153.74 ± 24.20	153.11 ± 23.85	0.805
DBP, mm Hg, mean ± SD	87.68 ± 13.03	87.89 ± 13.28	87.43 ± 12.75	0.742
Baseline NIHSS, mean ± SD	9.18 ± 7.07	6.16 ± 4.75	12.81 ± 7.69	< 0.001
Hgb, g/L, mean ± SD	136.38 ± 62.78	141.11 ± 82.70	130.67 ± 20.23	0.119
PLT, 10^9^/L, mean ± SD	211.25 ± 84.67	211.08 ± 78.28	211.46 ± 92.02	0.967
WBC, 10^9^/L, mean ± SD	8.65 ± 3.87	7.89 ± 3.84	9.58 ± 3.72	< 0.001
Neu, 10^9^/L, mean ± SD	6.47 ± 4.08	5.52 ± 2.29	7.61 ± 5.30	< 0.001
Ly, 10^9^/L, mean ± SD	1.49 ± 0.81	1.50 ± 0.58	1.47 ± 1.02	0.801
Mn, 10^9^/L, mean ± SD	0.61 ± 0.63	0.49 ± 0.21	0.75 ± 0.88	< 0.001
N/M, mean ± SD	5.81 ± 6.50	4.47 ± 3.13	7.42 ± 8.77	< 0.001
L/M, mean ± SD	3.14 ± 2.65	3.59 ± 2.82	2.61 ± 2.33	0.001
Eon, 10^9^/L, mean ± SD	0.13 ± 0.18	0.14 ± 0.19	0.11 ± 0.17	0.206
TC, mmol/L, mean ± SD	4.62 ± 1.17	4.39 ± 0.90	4.88 ± 1.39	< 0.001
TG, mmol/L, mean ± SD	1.63 ± 1.29	1.53 ± 1.12	1.74 ± 1.45	0.11
HDL, mmol/L, mean ± SD	1.24 ± 0.49	1.16 ± 0.34	1.35 ± 0.60	< 0.001
LDL, mmol/L, mean ± SD	2.83 ± 0.94	2.78 ± 0.83	2.90 ± 1.05	0.228
BUN, mmol/L, mean ± SD	6.42 ± 3.62	6.19 ± 3.75	6.70 ± 3.45	0.19
Cr, μmol/L, mean ± SD	83.96 ± 55.50	81.45 ± 57.57	86.98 ± 52.93	0.351
Glu, mmol/L, mean ± SD	7.74 ± 3.30	6.98 ± 2.65	8.66 ± 3.75	< 0.001
HbA1c, %, mean ± SD	8.19 ± 7.45	6.83 ± 2.00	9.82 ± 10.63	< 0.001
INR, mean ± SD	1.01 ± 0.12	0.99 ± 0.13	1.04 ± 0.12	< 0.001
FDPs, μg/mL, mean ± SD	7.00 ± 14.23	4.26 ± 8.68	10.30 ± 18.36	< 0.001
D-Dimer, mg/L, mean ± SD	2.73 ± 6.03	1.61 ± 3.45	4.09 ± 7.91	< 0.001
NT-proBNP, mean ± SD	1048.42 ± 2026.11	628.03 ± 1434.93	1554.97 ± 2475.16	< 0.001
*TOAST types, n (%)*				< 0.001
LAA	210 (59.2)	129 (66.5)	81 (50.3)	
CE	106 (29.9)	36 (18.6)	70 (43.5)	
SVO	29 (8.2)	25 (12.9)	4 (2.5)	
SOE	4 (1.1)	2 (1.0)	2 (1.2)	
SUE	6 (1.7)	2 (1.0)	4 (2.5)	

Non-HT, patients without hemorrhagic transformation; HT, patients with hemorrhagic transformation; Categorical variables are represented by the number (percent), and continuous variable are represented by mean (± standard deviation)

OTC, onset-to-CT time; SBP, systolic blood pressure; DBP, diastolic blood pressure; NIHSS, National Institute of Health Stroke Scale; Hgb, Hemoglobin; PLT, Blood platelet; WBC, white blood cell; Neu, neutrophil; Ly, lymphocyte; Mn, monocyte; N/M, neutrophil-monocyte ratio; L/M, lymphocyte-monocyte ratio; Eon, eosinophils; TC, total cholesterol; TG, triglycerides; HDL, high-density lipoprotein cholesterol; LDL, low-density lipoprotein cholesterol; BUN, blood urea nitrogen; Cr, Creatinine; Glu, Glucose; HbA1c, glycated hemoglobin A1c; INR, international normalized ratio; FDPs, fibrin degradation products; NT-proBNP, *N*-terminal pro-brain natriuretic peptide, ng/L; LAA, large artery atherosclerosis; SVO, small vessel occlusion; CE, cardioembolism; SOE, stroke of other determined etiology; SUE, stroke of undetermined etiology

**Fig. 4:**
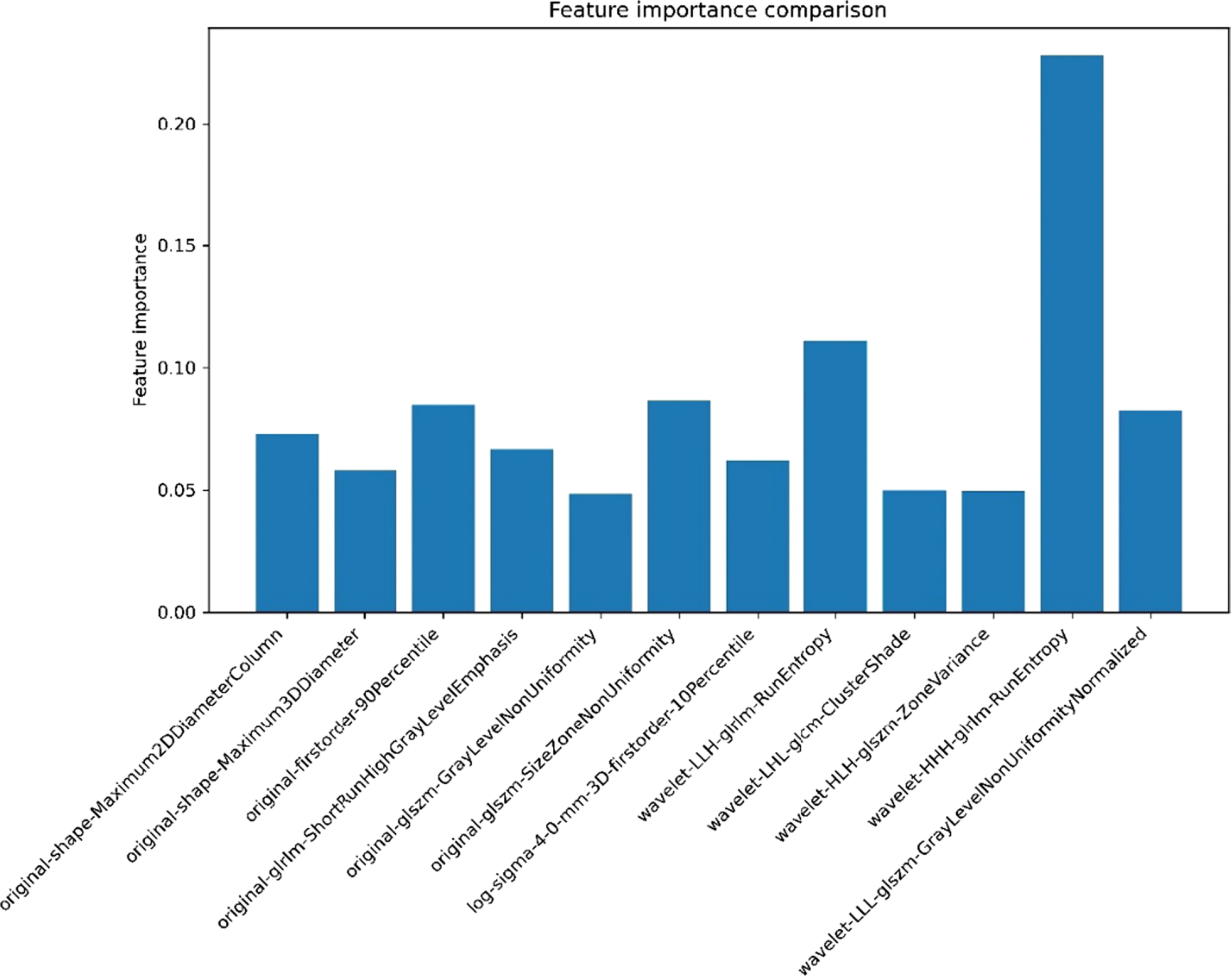
Importance of the 12 radiomics features

In all cases, 778 radiomics features were reduced to avoid model overfitting by RFE. Then, 12 radiomics features were selected for building radiomics model (Fig. 4).

### Performances of the clinical and radiomics models

The AUCs were calculated to assess the performance of the three models.

**Fig. 5:**
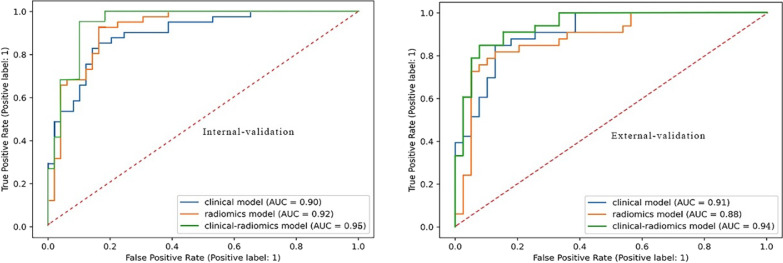
ROCs of the clinical model, radiomics model, and clinical–radiomics model by XGB (XGB, extreme gradient boosting)

The clinical model demonstrated an AUC of 0.996 (95% CI 0.991–0.999) in the training cohort, 0.898 (95% CI 0.873–0.921) in the internal validation cohort, and 0.911 (95% CI 0.891–0.928) in the external validation cohort for differentiating patients with HT (Fig. 5, Table 6).

**Table 6:** Performance of the three models

Model	Training cohort						Internal validation cohort						External validation cohort					
	AUC	ACC	SEN	SPE	PPV	NPV	AUC	ACC	SEN	SPE	PPV	NPV	AUC	ACC	SEN	SPE	PPV	NPV
Clinical	0.996	0.969	0.981	0.959	0.952	0.984	0.898	0.844	0.854	0.837	0.814	0.872	0.911	0.833	0.788	0.872	0.839	0.829
Radiomics	0.999	0.972	0.994	0.954	0.947	0.995	0.922	0.878	0.963	0.837	0.826	0.932	0.883	0.847	0.727	0.949	0.923	0.804
Combined	0.995	0.980	0.994	0.9691	0.964	0.995	0.950	0.900	0.951	0.857	0.848	0.945	0.942	0.861	0.758	0.949	0.926	0.822

In the training cohort, the radiomics model displayed an AUC of 0.999 (95% CI 0.999–1.000); internal validation cohort, 0.922 (95% CI 0.896–0.941); and external validation cohort, 0.883 (95% CI 0.851–0.902) (Fig. 5, Table 6).

### Performances of the clinical-radiomics model

In the training cohort, the AUC of this clinical–radiomics model was 0.995 (95%CI 0.991–0.999); in the internal validation cohort, it was 0.950 (95% CI 0.925–0.967), and in the external validation cohort, it was 0.942 (95% CI 0.927–0.958) (Fig. 5, Table 6). The DeLong test demonstrated no difference between the clinical model and the clinical–radiomics combination model in the training cohort, internal validation cohort or external validation cohort (*p* = 0.954, 0.179, and 0.364, respectively).

**Fig. 6:**
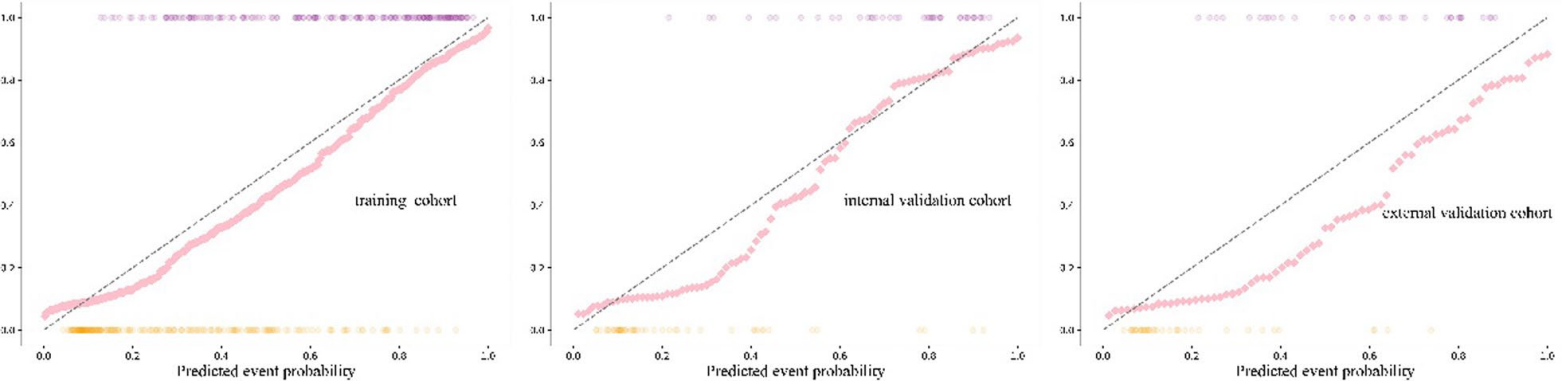
Calibration curves for the clinical–radiomics model in the training and validation cohorts

In the training cohort (*p* = 0.458) and internal validation cohort (*p* = 0.341), the clinical–radiomics model and the observed result had excellent agreement on the calibration curve for the potential of HT. However, the external validation cohort (*p* = 0.032) had slightly worse consistency (Fig. 6).

**Fig. 7:**
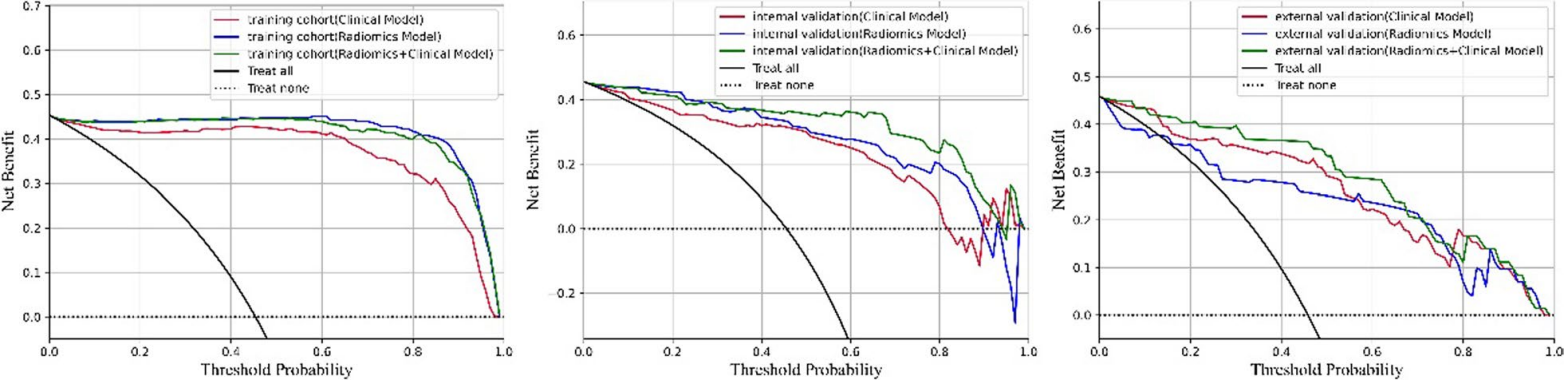
DCA for the clinical, radiomics, and clinical–radiomics model in the training and validation cohorts (DCA, Decision Curve Analysis)

The decision analysis curves for all three models indicated that they were all clinically useful in predicting the HT (Fig. 7).

## Discussion

In this study, we developed an ML approach by incorporating clinical data with radiomics-based features from NCCT to predict the risk of HT postthrombolysis. It compensates for the limitations of visual recognition of NCCT image signs, reduces misdiagnosis and missed diagnosis by inexperienced first-line doctors, and increases their diagnostic confidence. By contrast, because these clinical data are obtained by routine examinations after admission, this model can be applied rapidly and is practical. In addition, we used data from multiple centers for modeling and independent external verification, so the model has a high degree of generalizability.

In this study, five clinical variables including NIHSS, FDPs, D-dimer, monocytes, and NT-proBNP on admission were found to be significant risk factors of HT for patients with AIS undergoing IVT. NIHSS is frequently used to measure the severity of a stroke, as it is effective for determining awareness and motor, sensation, response, and advanced nerve functioning [27]. Previous studies have shown that NIHSS was an independent risk factor of postthrombolysis HT [28, 29], which is consistent with the finding of our study. This indicates the need to avoid thrombolytic therapy if a patient suffers from a serious stroke. A study demonstrated that the FDPs on admission were related to PH [6, 30] because FDPs might impede platelet aggregation by competing with fibrinogen for binding to the platelet membrane [31, 32]. D-dimers may cause monocytes to produce and release proinflammatory cytokines, such as interleukin-6 (IL-6) and IL-1β [33], which will augment the HT risk [34]. These findings suggest that the coagulation index is very important for predicting HT, which needs the close attention of doctors. Finally, NT-proBNP is an inactive fragment derived from the cleavage of BNP [35]. In line with earlier studies [36–38], our findings showed that NT-proBNP level was independently associated with HT in patients with stroke who had received IVT. One possible explanation was that HT exacerbated the brain damage caused by ischemic stroke [39]. NT-proBNP needs to be distinguished from other disorders, such as heart failure; when its level increases, its sensitivity is great, but its specificity is weak. Thus, if a patient suffers from AIS, the doctor may recognize that the patient has a higher risk of HT.

The proposed clinical model could be utilized to estimate the HT risk and had excellent prediction efficiency. In this study, the AUC of the clinical model in the external validation was slightly greater than that in the internal validation, demonstrating a stronger capacity for generalization. Previous studies have only used clinical baseline data to predict HT [24], which is best suited for patients without access to imaging data. Since HT is primarily from the infarction itself and it is not sufficient to analyze merely clinical signs, we extracted the imaging characteristics of the ischemic area.

Then, the features extracted from NCCT images were used to develop the radiomics model, and the prediction efficiency was also great. In this study, 12 optimal quantitative radiomics features were extracted. Among them, the wavelet-HHH-glrlm-RunEntropy was the most important feature that mirrored the apparent heterogeneity of the infarction. The result might imply that the HT risk increases with the degree of heterogeneity of the infarct area. Nevertheless, further studies are required to elucidate the relationship between the pathological changes in HT and NCCT-based radiomics features. In addition, the shape features described the size of the cerebral infarction. The findings were similar to those of earlier studies [40] and revealed that the larger the infarct zone, the greater the HT risk. Consequently, although some signs on NCCT, such as the hyperdense middle cerebral arteries sign or the Alberta Stroke Program Early CT Score, can predict the risk of HT [41], front-line doctors may fail to recognize these signals because they lack experience, with which could be compensated by our radiomics-based models.

In this study, although DeLong's method yielded no statistical significance, it is noteworthy that AUC and accuracy were consistently higher in clinical-radiomics model than clinical model, across internal and external validation sets, showing the potential for radiomics to improve the prediction for future HT. In addition, the DCA results also revealed that the three models had a significant net benefit in predicting HT. As a result, if these models will be used as trustworthy, repeatable tools can guide therapeutic decisions. Moreover, they are less time-consuming—the model will actually identify the HT risk of a new patient within only a minute. Thus, this model may be used in clinical practice as soon as possible after being confirmed by a larger group.

This study has some limitations. First, because this was a retrospective study with a limited sample size, although participants from seven hospitals were included, some selection biases may have occurred. Moreover, because part of clinical data of some patients was incomplete, K-nearest neighbor method was used to fill in the missing values, which was the limitation of the study. After this study’s attempt, the NCCT-based model and clinically relevant data were quite efficient, which gave us more confidence to broaden the scope of our subsequent research. Later, despite studies on radiomics nowadays, including those on tumor, inflammation, and cerebrovascular illness, the model’s interpretation of radiomics features is generally limited or incomplete. Therefore, it is expected that subsequent research would gradually shed light on the internal link between radiomics features and clinical results. Furthermore, the intrinsic attribute of CT radiomics makes that most radiomic features are highly affected by CT acquisition and reconstruction settings [42], so we preprocessed all the NCCT images to reduce the impact of these factors. Thirdly, this study's reproducibility and viability may be limited by the fact that the ROI from post-IVT MRI was not feasible in a setting where only pre-IVT NCCT was administered. Fourthly, this article only predicts whether HT will occur, not including HT typing (HI and PH). Finally, some risk factors related to HT were not included, such as matrix metalloproteinases, homocysteine, and others, because some hospitals lacked the necessary laboratory indices. In actuality, many multicenter or big data research projects also struggle with this challenging issue. The integrity and consistency of the current data must be resolved because doctors in different institutions cannot agree on the same disease examination method and equipment, and patient’s compliance is different. As a result, more institutions must join the project of predicting HT, which calls for more alluring experts to organize them to do so. In the future, increasing their clinical practicability could help in integrating more clinical characteristics into the model and expand the number of samples.

## Conclusion

By using radiomics features extracted from NCCT images and clinical features, this study established a model that demonstrated great performance and individualized risk assessment of HT for patients who received IVT. This trustworthy model can help first-line doctors identify patients who are at a significantly higher risk of HT and support them when they make clinical decisions.

## Supplementary Information

**Additional file 1. Table S1.** Models of CT Scanners and Scanning Parameters for Seven Institutions. **Figure S1.** Performance of different ICC thresholds in the internal validation.

## Data Availability

The datasets generated and/or analyzed during the current study are not publicly available because the subjects did not provide written consent for their data to be publicly shared.

## Abbreviations

ACC: Accuracy
AIS: Acute ischemic stroke
AUC: Area under the receiver operating characteristic curve
BUN: Blood urea nitrogen
CE: Cardioembolism
Cr: Creatinine
DBP: Diastolic blood pressure
DCA: Decision curve analysis
Dim: Dimension
Eon: Eosinophils
FDPs: Fibrin degradation products
GLCM: Gray-level co-occurrence matrix
GLRLM: Gray-level run length matrix
GLSZM: Gray-level size zone matrix
Glu: Glucose
HbA1c: Glycated hemoglobin A1c
HDL: High-density lipoprotein cholesterol
Hgb: Hemoglobin
HI: Hemorrhagic infarction
HT: Hemorrhagic transformation
ICC: Intraclass correlation coefficient
INR: International normalized ratio
IVT: Intravenous thrombolysis
L/M: Lymphocyte-monocyte ratio
LAA: Large artery atherosclerosis
LASSO: Least absolute shrinkage and selection operator
LDL: Low-density lipoprotein cholesterol
Linear SVC: Linear support vector classification
LR: Logistic regression
Ly: Lymphocyte
ML: Machine learning
Mn: Monocyte
MRI: Magnetic resonance imaging
N/M: Neutrophil-monocyte ratio
NCCT: Noncontrast computed tomography
Neu: Neutrophil
NIHSS: National Institute of Health Stroke Scale
NPV: Negative predictive value
NT-proBNP: *N*-Terminal pro-brain natriuretic peptide
OTC: Onset-to-CT time
PH: Parenchymal hemorrhage
PLT: Blood platelet
PPV: Positive predictive value
RF: Random forest
RFE: Recursive feature elimination
RFECV: Recursive feature elimination cross validation
ROI: The region of interest
SBP: Systolic blood pressure
SEN: Sensitivity
SGD: Stochastic gradient descent
SOE: Stroke of other determined etiology
SPE: Specificity
SUE: Stroke of undetermined etiology
SVM: Support vector machine
SVO: Small vessel occlusion
TC: Total cholesterol
TG: Triglycerides
TOAST: Trial of ORG 10172 in acute stroke treatment
WBC: White blood cell
XGB: EXtreme gradient boosting
