# Supplementary material for: A clinical–radiomics model based on noncontrast computed tomography to predict hemorrhagic transformation after stroke by machine learning: a multicenter study
Source: Insights Imaging. 2023 Mar 29;14:52. doi: 10.1186/s13244-023-01399-5 (PMC10050271; doi:10.1186/s13244-023-01399-5)
Supplement: Supplementary file 1 — Additional file 1. Table S1. Models of CT Scanners and Scanning Parameters for Seven Institutions. Figure S1. Performance of different ICC thresholds in the internal validation. [file 13244_2023_1399_MOESM1_ESM.docx]

**Additional file**

**Materials and methods**

Imaging acquisition

Table S1 shows the models of CT Scanners and Scanning Parameters for Seven Institutions.

**Table S1.** Models of CT Scanners and Scanning Parameters for Seven Institutions

| Center | equipment | Tube voltage (KV) | Tube current (mA) | Matrix size | Slice thickness  (mm) | Slice  Spacing  (mm) |
| --- | --- | --- | --- | --- | --- | --- |
| 1 | Canon Aquilion one | 130 | 90-170 | 512×512 | 5 | 5 |
| 2 | Siments SOMATOM  difinition Flash | 120 | 150-190 | 512×512 | 5 | 5 |
| 3 | UNITED IMAGING uCT 760 | 120 | 160-230 | 512×512 | 5 | 5 |
| 4 | Philips Brilliance iCT | 120 | 200-240 | 512×512 | 5 | 5 |
| 5 | GE LightSpeed VCT | 120 | 230-300 | 512×512 | 5 | 5 |
| 6 | Siments SOMATOM  difinition Flash | 120 | 180-235 | 512×512 | 5 | 5 |
| 7 | GE BrightSpeed Elite | 120 | 170-200 | 512×512 | 5 | 5 |

Radiomics analysis

Comparing the ROIs’ features of radiologists 1 and 2 allowed for the calculation of the inter-ICC. The features (with both ICCs threshold ≥ 0.95) having good reliability were added to the subsequent analysis (Figure S1).


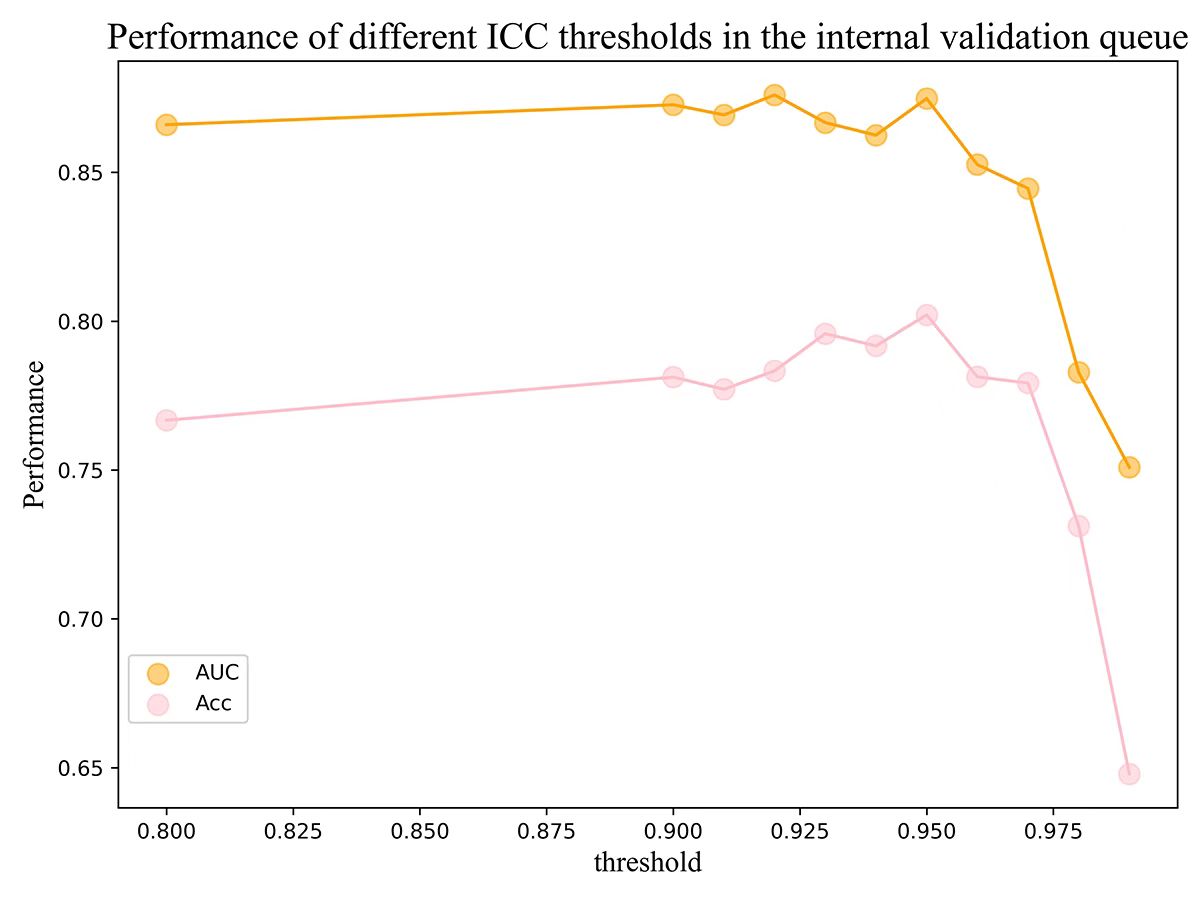


Figure S1 Performance of different ICC thresholds in the internal validation
